# Exenatide Protects Against Cardiac Dysfunction by Attenuating Oxidative Stress in the Diabetic Mouse Heart

**DOI:** 10.3389/fendo.2019.00202

**Published:** 2019-04-05

**Authors:** Wei Ding, Wen-guang Chang, Xiao-ci Guo, Ying Liu, Dan-dan Xiao, Dan Ding, Jian-xun Wang, Xue-juan Zhang

**Affiliations:** ^1^Department of General Medicine, The Affiliated Hospital, Qingdao University, Qingdao, China; ^2^Center for Regenerative Medicine, Institute for Translational Medicine, Qingdao University, Qingdao, China; ^3^School of Basic Medical Sciences, Qingdao University, Qingdao, China

**Keywords:** exenatide, diabetes, ROS, apoptosis, cardiac function

## Abstract

Cardiovascular disease is the major cause of death in patients with diabetes. Current treatment strategies for diabetes rely on lifestyle changes and glucose control to prevent angiopathy and organ failure. Exenatide, a glucagon-like peptide-1 (GLP-1) receptor agonist, is used as an add-on therapy to insulin treatment. Exenatide also has multiple beneficial effects in addition to its hypoglycemic effects, such as preventing hepatic steatosis and protecting against cardiac injury from doxorubicin-induced cardiotoxicity or ischemic reperfusion. However, the mechanisms underlying the cardioprotective effects of exenatide in diabetes have not been fully clarified. To address this issue, we investigated the cardioprotective effects of exenatide in type 1 and type 2 diabetic mice. We found that exenatide simultaneously attenuated reactive oxidative species (ROS) production through increases in the antioxidant enzymes manganese dependent superoxide dismutase (MnSOD) and catalase. Moreover, exenatide decreased tumor protein P53 (p53) expression and prevented cell apoptosis in H9c2 cells. The presence of the catalase inhibitor 3-AT attenuated the effects of exenatide. Overall, the results strongly indicate that exenatide treatment may be protective against the development of diabetic cardiomyopathy.

## Introduction

Diabetes mellitus is a metabolic disorder characterized by hyperglycemia. Cardiovascular disease is the major cause of death in diabetic patients. Patients with diabetes are at least 10 times more likely to suffer from heart dysfunction than their non-diabetic counterparts (1–3). Current treatment strategies for diabetes rely on lifestyle changes and glucose control to prevent angiopathy and organ failure. Although many hypoglycemic agents have been extensively investigated in recent years, there is still a need for effective therapeutic strategies for better results.

GLP-1 is an insulinotropic hormone released from intestinal L cells in response to nutrient ingestion, and it modulates glucose metabolism by affecting pancreatic islet secretions. However, it can be rapidly inactivated by the enzyme dipeptidyl peptidase IV in the circulation. Exenatide is a kind of GLP-1 analog that can evade rapid clearance by dipeptidyl peptidase IV; thus, it has a long half-life when injected subcutaneously. Exenatide was approved for type 2 diabetic treatment in 2005. Previous studies have shown that in addition to the hypoglycemic effect, exenatide reduced weight gain and alleviated hepatic steatosis in diabetic mice (4, 5). In addition, several studies demonstrated that exenatide has beneficial effects on cardiac function. In a clinical trial, a study was conducted on 23 patients with uncomplicated type 2 diabetes. Administration of exenatide (10 μg, bid) improved cardiac function and reduced arterial stiffness (6). Another clinical study also showed that exenatide twice daily as an add-on therapy was associated with significant, sustained improvement in several cardiovascular risk markers in patients with type 2 diabetes vs. glimepiride treatment (7). In addition, adjunctive exenatide administered with primary percutaneous coronary intervention was shown to reduce infarct size and improve subclinical left ventricular function in patients with ST segment elevation myocardial infarction (8). Consistent with these findings, in animal studies, streptozotocin-induced diabetic rats that underwent a 12-week treatment with exenatide exhibited significantly improved cardiac function, glucose uptake, and microvascular barrier function (9). Exenatide pretreatment improved cardiac function and reduced the infarct area in rat hearts after ischemia reperfusion (10). Additionally, exenatide protects cardiomyocytes against doxorubicin-induced cardiotoxicity (11).

The pathological mechanisms underlying diabetic cardiomyopathy, including inflammation, mitochondria dysfunction, lipotoxicity, and oxidative stress, are complex and multifactorial. The molecular mechanisms underlying the cardioprotective effects of exenatide remain unclear. It has been reported that exenatide prevents cardiomyocyte apoptosis by enhancing autophagy and reducing lipotoxic ceramide accumulation (12, 13), as well as preventing mitochondrial dysfunction and endoplasmic reticulum stress (14, 15). Oxidative stress and accelerated reactive oxidative species (ROS) production induced by high glucose are known to play key roles in the progression of diabetic cardiovascular disease and cardiomyocyte apoptosis. We hypothesize that exenatide could exert its cardioprotective effects by preventing ROS production (16–18). Thus, this study was designed to determine the protective effects of exenatide on cardiac functions in mouse models of both type 1 and type 2 diabetes and to explore the underlying molecular mechanism. *In vivo* and *in vitro* studies were performed to assess cardiac function, glucose tolerance, antioxidative stress capacity and cell apoptosis. The effects of exenatide on ROS production and apoptosis were evaluated in H9c2 cardiomyoblasts. Furthermore, the underlying mechanism of exenatide-induced protective effects was investigated. Our study provides a new mechanism underlying the cardioprotective actions of exenatide.

## Materials and Methods

### Animal Model

Six-week-old male C57BL/6J mice were purchased from Beijing Vital River Laboratory Animal Technology. The mice were housed in an environmentally controlled breeding room (temperature: 20 ± 2°C, humidity: 60 ± 5%, 12/12 light-dark cycle). All mice had free access to tap water. All procedures were approved by the Ethics Committee for the Use of Experimental Animals of Qingdao University.

### Type 2 Diabetic Mice

For the high-fat diet-induced type 2 diabetic mouse study, mice were randomly distributed into two initial groups fed a control diet (10% Kcal fat, 70% Kcal carbohydrate, and 23% Kcal protein with a total caloric value of 3.85 Kcal/gm. Research diet D12450B, CON group, *n* = 8) or a high-fat diet (consisting of 45% Kcal fat, 35% Kcal carbohydrate, and 20% Kcal protein with a total caloric value of 4.73 Kcal/gm. Research diet D12451 HFD group, *n* = 16). After a 20-week dietary intervention, the HFD group was then randomly subdivided to receive either exenatide (24 nmol/kg/d, BYETTA; T2DM+EXE group, *n* = 8) or normal saline as a control (T2DM group, *n* = 8) by intraperitoneal injection during the light cycle while continuing the HFD feeding for 4 weeks.

### Type 1 Diabetic Mice

Type 1 diabetes was induced by treating the mice with a high dose of streptozotocin (STZ) (Sigma-Aldrich, Shanghai, China) as previously described (19). Briefly, the mice received an intraperitoneal injection of STZ at a dose of 120 mg/kg (dissolved in 0.1 mol/L citrate buffer, pH 4.5). To observe the diabetic status of the mice, the non-fasting glucose level was monitored from tail blood samples by a glucose meter (Roche, ACCU-CHEK, Performa glucometer). Diabetes onset was diagnosed when the blood glucose level was >16.7 mmol (300 mg/dl) on 2 consecutive tests. Then, the type 1 diabetic mice were randomly separated into two groups that were both fed a control diet. The mice then received either exenatide (24 nmol/kg/d, BYETTA; T1DM+EXE group, *n* = 8) or normal saline as a control (T1DM group, *n* = 8) by intraperitoneal injection during the light cycle while continuing feeding with the chow diet for 4 weeks.

### Intraperitoneal Glucose Tolerance Test (IPGTT)

After 24 weeks, body weights were measured in all groups. However, the glucose tolerance test (*n* = 8 in each group) was measured only in type 2 diabetic mice. Briefly, the IPGTT was conducted after an overnight fast (12–16 h). Mice were injected with 40% glucose (2 g/kg body weight, i.p.). Blood glucose was measured from the tail tip using a glucose meter (Roche, ACCU-CHEK, Performa glucometer) at 0, 30, 60, 90, and 120 min.

### HOMA-IR Measurement

Plasma insulin (*n* = 8 in each group) was measured using a commercial ELISA kit (Beyotime, China). Homeostasis model assessment (HOMA-IR) was calculated by fasting plasma glucose (FPG, mmol/L) × fasting plasma insulin (FINS, mIU/L)/22.5.

### Echocardiography

After 24 weeks, the mice in each group were anesthetized using 0.3% pentobarbital sodium (intraperitoneal injection). Transthoracic two-dimensional M-mode echocardiography and pulsed wave Doppler spectral tracings were obtained using a Vevo 2100 Imaging System (VisualSonics Inc., Canada) with a 30-MHz transducer for each mouse. The percentages of ejection fraction (EF%), left ventricular end-systolic diameter (LVSD), and left ventricular end-diastolic diameter (LVDD) were measured using M-mode tracings. The percentage of fractional shortening (FS%) was calculated according to the following formula [(LVDD-LVSD)/LVDD] × 100%. The ratio of early ventricular filling peak velocity (E wave) and late filling velocity (A wave) (E/A) were measured by pulsed wave Doppler spectral tracings to determine the diastolic function.

### Cell Culture

H9c2 cardiomyoblasts were purchased from Cobioer (Nanjing, China, ATCC origin). H9c2 cells were maintained in high-glucose Dulbecco's modified Eagle's medium (DMEM, glucose 25 mM) with 10% fetal bovine serum (FBS), 2% L-glutamine, 10% sodium bicarbonate, 10% sodium pyruvate, 5% HEPES, 1% penicillin/streptomycin, and 1% gentamycin in an incubator (37°C, 5% CO2). For high-glucose treatment, H9c2 cells were incubated for 24 h in high-glucose medium (40 mM) with or without exenatide present (25 nM, 50 nM).

### TUNEL Assay

The terminal deoxynucleotidyl transferase-mediated deoxyuridine triphosphate nick-end labeling (TUNEL) assay was carried out according to the manufacturer's protocol with a one-step TUNEL apoptosis assay kit (Beyotime, Shanghai, China). The results were scored semiquantitatively by averaging the numbers of TUNEL-positive cells per low-power field for five fields in each experiment.

### ROS Determination

H9c2 cells in the presence of control medium or high-glucose medium (40 mM) were treated with or without exenatide (25 or 50 nM) in the dark with 10 μM DCFH-DA (Beyotime Biotechnology, Shanghai, China) for 20 min. To observe DCF fluorescence during illumination, cells were mounted on a microscope slide and illuminated and visualized with a 20 × objective on a Nikon-TI SR microscope. The number of positive cells was counted in at least three random pictures in each group.

To further quantify ROS production, H9c2 cells were seeded in 96-well plates in the presence of high glucose and treated with or without exenatide (25 or 50 nM). After 10 μM DCFH-DA labeling, the fluorescence intensity of the cells was recorded by a fluorescence microplate reader at 488/525 nm.

### Western Blotting Analysis

Hearts from each group of animals were homogenized, and protein samples were prepared with ice-cold RIPA lysis buffer. Protein concentrations were measured using the Bradford assay (Bio-Rad protein assay kit). For western blotting detection of p53, catalase, MnSOD, and glyceraldehyde 3-phosphate dehydrogenase (GAPDH) protein expression, 80 μg of protein was used. All proteins were separated by 12% SDS polyacrylamide gel and electrotransferred onto polyvinylidene fluoride membranes (PVDF). Membranes were blocked with 5% (w/v) skim milk for 2 h at room temperature and then incubated with primary antibodies with gentle agitation overnight at 4°C. The membranes were washed 3 times for 10 min each with 15 ml of TBST (10 mM Tris–HCl, 150 mM NaCl and 0.1% (v/v) Tween-20 and then incubated with secondary antibody at room temperature for 2 h.

For *in vitro* western blotting experiments, upon completion of the experiments, H9c2 cells were harvested using ice-cold RIPA lysis buffer. Protein concentrations were measured using the Bradford assay (Bio-Rad protein assay kit). For western blotting detection of P53, catalase and MnSOD protein expression, the method used is the same as that described above.

Proteins for *in vivo* and *in vitro* experiments were visualized with enhanced chemiluminescence solution and images generated with a GENE Imaging system. The images were quantified using Image Analysis Software (Quantity One). Primary antibodies for p53 (catalog number, 60283-2-Ig), catalase (catalog number, 21260-1-AP), and MnSOD (catalog number, 66474-1-Ig) were purchased from Proteintech (Proteintech Group, Inc., USA). GAPDH and HRP-conjugated secondary antibodies were purchased from Yesen Biotechnology (Shanghai, China).

### Statistical Analysis

Data are presented as the mean ± SEM. An unpaired two-tailed Student's *t*-test was used to determine significant differences between groups. For comparison of more than two groups, one-way analysis of variance (ANOVA) followed by Tukey's *post hoc* test was performed. Adjusted two-sided *P*-values were calculated, and *P* < 0.05 was considered indicative of statistical significance.

## Results

### Exenatide Treatment Reduces Body Weight and Attenuates Glucose Intolerance in Type 2 Diabetic Mice

**Figure 1:**
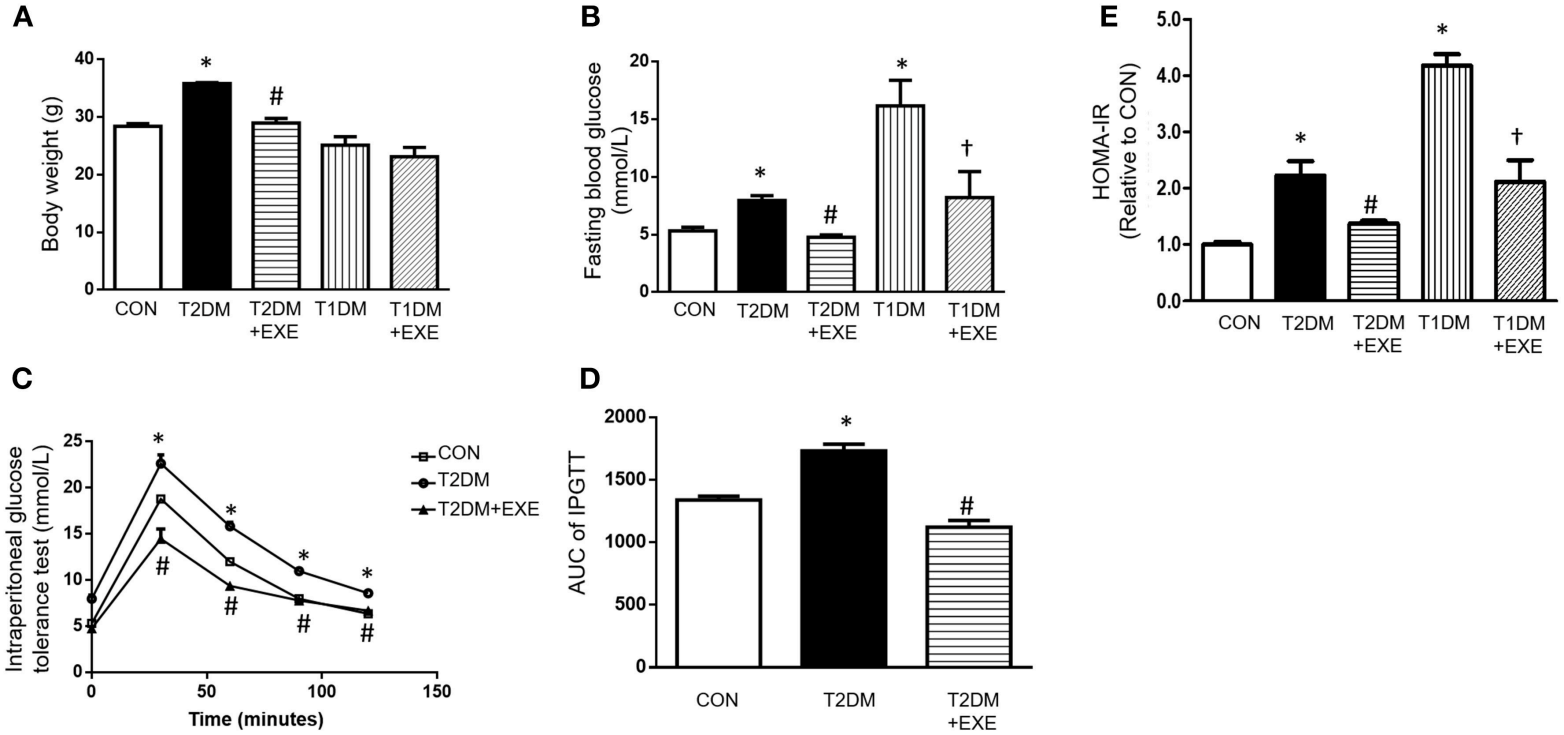
Effects of exenatide on body weight and glucose tolerance in type 1 and type 2 diabetic mice. Type 1 and type 2 diabetic models were constructed as described in the section Materials and Methods. Either saline or exenatide (24 nmol/kg/d) was injected intraperitoneally daily for 4 weeks. Body weight **(A)**, fasting blood glucose levels **(B)**, intraperitoneal glucose tolerance test (IPGTT) results **(C)**, area under curve for IPGTT **(D)** and HOMA-IR levels **(E)**. Values are the mean ± S.E.M. of 7–10 animals per group. ^*^p < 0.05 vs. control group, ^#^*p* < 0.05 vs. T2DM group, ^†^*p* < 0.05 vs. T1DM group.

The mean body weights of the CON, T2DM, T2DM+EXE and T1DM, T1DM+EXE animals were measured. The body weight of T2DM mice was increased compared to that of the CON mice, and treatment of T2DM animals with exenatide (24 nmol/kg/d) decreased body weight significantly. T1DM mice had significantly reduced body weight compared to the CON mice, and treatment of T1DM animals with exenatide (24 nmol/kg/d) slightly increased body weight, although the difference was not significant (Figure 1A). In addition, fasting blood glucose (FBG) levels in both T1DM and T2DM groups was increased compared to that in the CON group, and exenatide-treated mice showed decreased FBG levels compared to model animals (Figure 1B). Moreover, the intraperitoneal glucose tolerance test and Homeostasis model assessment (HOMA-IR) levels showed that exenatide improved glucose tolerance, insulin resistance and reduced the area under the curve (AUC) in T2DM animals (Figures 1C–E).

### Exenatide Protects Against Cardiac Dysfunction in Diabetic Mice

**Figure 2:**
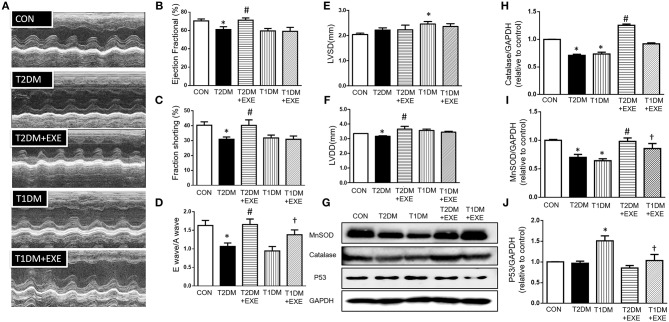
Echocardiographic analyses of CON, T2DM, T1DM, T2DM+EXE, and T1DM+EXE mice. Cardiac function was measured by echocardiography 1 week prior to sacrifice as described in the Materials and Methods. M-mode cardiographs **(A)**, Percent ejection fraction (EF%) **(B)**, Percent left ventricular fractional shortening (FS%) **(C)**, Mitral valvular inflows showing E wave and A wave (E wave: LV early-filling wave; A wave: filling from atrial contraction). Ratio of the early to late peak diastolic trans-mitral flow velocity (E/A) **(D)**. Left ventricular internal dimension-systolic (LVSD) **(E)**. Left ventricular internal dimension-diastolic (LVDD) **(F)**. Data represent the mean ± S.E.M. (*n* = 8 mice per group), ^*^p < 0.05 vs. control group, ^#^*p* < 0.05 vs. T2DM group, ^†^*p* < 0.05 vs. T1DM group. MnSOD, catalase and p53 levels in mouse heart **(G–J)**. Data represent the mean ± S.E.M., and at least three samples were used for the western blotting analysis. ^*^p < 0.05 vs. control group, ^#^*p* < 0.05 vs. T2DM group, ^†^*p* < 0.05 vs. T1DM group.

To assess whether exenatide protects against the development of diabetic cardiomyopathy in diabetic mice, echocardiography was performed on CON, T2DM, T2DM+EXE and T1DM, T1DM+EXE animals one week prior to sacrifice. EF%, LVSD, and LVDD were used to determine systolic function, and FS% and E/A were used to determine diastolic function. Systolic and diastolic dysfunction developed in T2DM and T1DM animals in comparison to CON mice, and exenatide treatment of T2DM animals attenuated this dysfunction (Figure 2A). LVSD was increased in the T1DM group compared to CON group; however, there was no significant difference between the T2DM and CON groups (Figure 2E), and LVDD was decreased in the T2DM group compared to the CON group (Figure 2F). E/A, FS, and EF% were all significantly decreased in T2DM and T1DM animals compared to CON mice. Treatment of T2DM animals with exenatide restored LVDD, E/A, FS, and EF% to that of CON animals (Figures 2B–D,F). Moreover, treatment of T1DM animals with exenatide restored only E/A to that of CON animals (Figure 2D). Thus, exenatide treatment attenuates both systolic and diastolic dysfunction in type 2 diabetic mice and attenuates only diastolic dysfunction in T1DM mice.

### Exenatide Treatment Increases Cardiac MnSOD and Catalase and Reduces P53 in Diabetic Mouse Heart

It was previously demonstrated that exenatide treatment attenuated myocardial infarction-induced cardiac injury through reduced ROS production (20). MnSOD and catalase are important enzymes that protect the cell from oxidative damage. We examined MnSOD and catalase levels in CON, T2DM, T2DM+EXE, T1DM, and T1DM+EXE animals. The levels of MnSOD and catalase protein were significantly reduced in T2DM and T1DM animals compared to CON mice. Exenatide-treated T2DM and T1DM animals showed significant increases in MnSOD and catalase compared to model animals (Figures 2G–I).

P53 is known to initiate apoptosis upon DNA damage. We examined cardiac p53 levels in CON, T2DM, T2DM+EXE, T1DM, and T1DM+EXE animals. The level of p53 protein was significantly increased in T2DM and T1DM animals compared to CON mice. Exenatide-treated T2DM and T1DM animals exhibited significantly decreased p53 levels compared to diabetic animals (Figures 2G,J). Thus, exenatide treatment activates cardiac antioxidative activity and reduces P53 activity in diabetic cardiomyopathy mice.

### Exenatide Treatment Attenuates High Glucose-Induced ROS Production in H9c2 Cardiomyoblasts by Increasing Antioxidant Enzymes

H9c2 myoblasts are a cell model used as an alternative for cardiomyocytes. It has been widely used for diabetes-related studies *in vitro* (21, 22). Therefore, we used this cell line to investigate the molecular mechanisms underlying the protective effects of exenatide.

**Figure 3:**
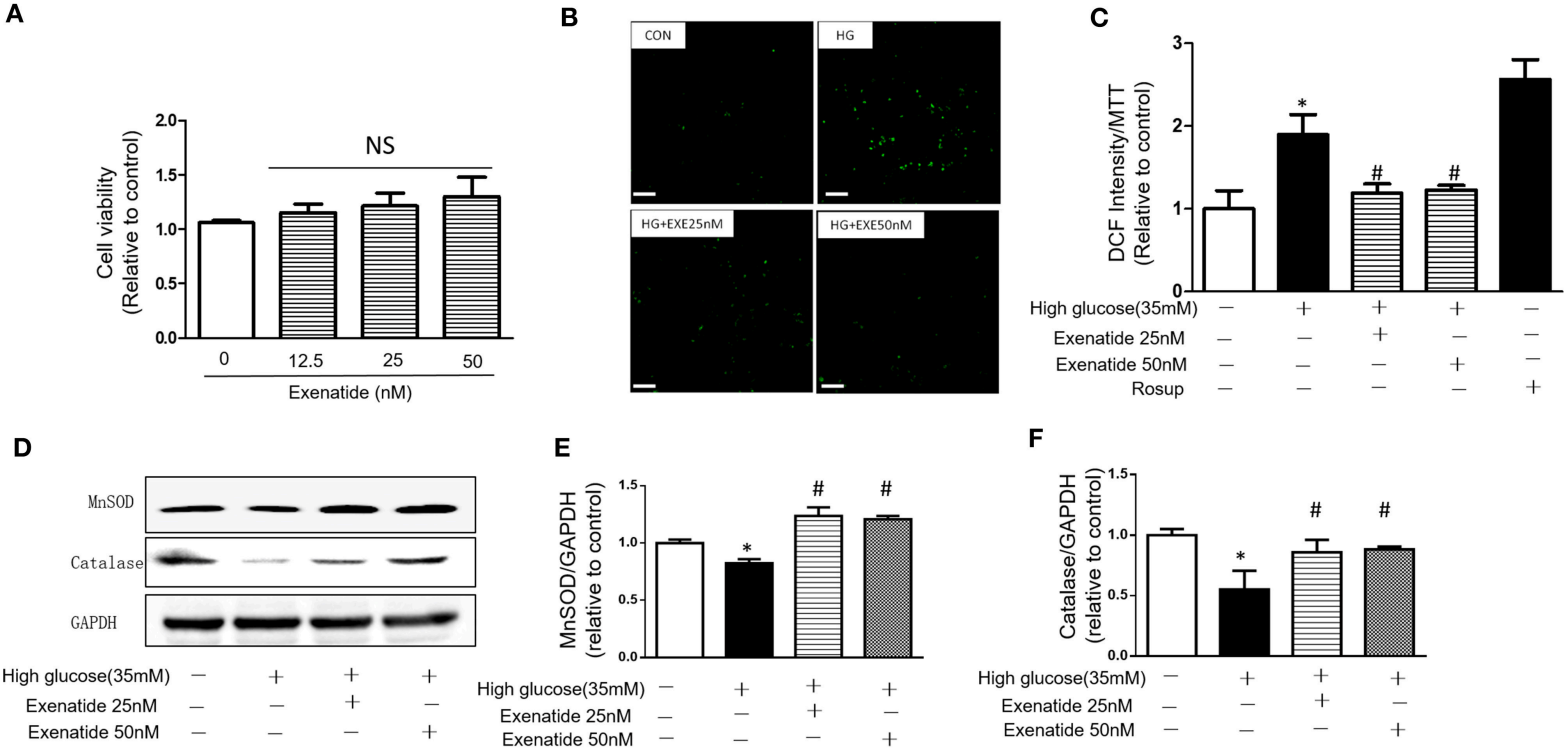
Effects of exenatide on ROS production and antioxidant enzymes in high glucose-induced H9c2 cells. H9c2 cells were incubated with 0–50 nM concentrations of exenatide, and cell viability was determined as described in the Materials and Methods. Cell viability **(A)**. H9c2 cells were incubated with 35 mM glucose in the presence or absence of exenatide (25 or 50 nM), and ROS production was determined as described in the Materials and Methods. Representative ROS fluorescence intensities are depicted; white scale bar, 50 μm **(B)**. A separate experiment for ROS production measured by the fluorescence intensity of cells using a fluorescence microplate reader at 488/525 nm **(C)**. H9c2 cells were incubated with 35 mM glucose in the presence or absence of exenatide (25 or 50 nM), and MnSOD, catalase and p53 levels were measured by western blotting as described in the Materials and Methods. A representative micrograph is depicted **(D)**, MnSOD expression **(E)** catalase expression **(F)**. Data represent the mean ± S.E.M. The data represent at least three separate experiments, and each group has at least 3 samples. ^*^p < 0.05 vs. control group, ^#^*p* < 0.05 vs. high-glucose group.

H9c2 cardiomyoblasts were incubated for 24 h with various concentrations of exenatide (0–50 nM), and cytotoxicity was examined. Incubation of cells with 0–50 nM exenatide was not cytotoxic (Figure 3A).

To further determine the effects of exenatide on oxidative stress induced by high glucose, the ROS levels were measured after exenatide treatment (25 or 50 nM) in the presence of high glucose (35 mM) in H9c2 cells. ROS levels were significantly increased by high-glucose stimulation compared with those in the control group (*P* < 0.05). Exenatide at concentrations of 25 and 50 nM for 24 h decreased the ROS level (Figure 3B). The results were consistent with a qualified test of ROS, which was measured by the fluorescence intensity of cells by a fluorescence microplate reader at 488/525 nm (Figure 3C). Rosup was used as a positive control.

Then, levels of the antioxidant enzymes MnSOD and catalase were measured in H9c2 cells after exenatide (25 or 50 nM) treatment in the presence of high glucose (35 mM). MnSOD and catalase levels in the high-glucose group were significantly decreased compared with those in the control group (*P* < 0.05). Exenatide treatment at concentrations of 25 and 50 nM for 24 h increased the MnSOD (*P* < 0.05) and catalase levels (*P* < 0.05) (Figures 3D–F). These results indicated that exenatide reduced oxidative stress induced by high glucose by increasing the concentration of antioxidant defense enzymes in H9c2 cells.

### Exenatide Treatment Attenuates High Glucose-Induced Apoptosis in H9c2 Cells

**Figure 4:**
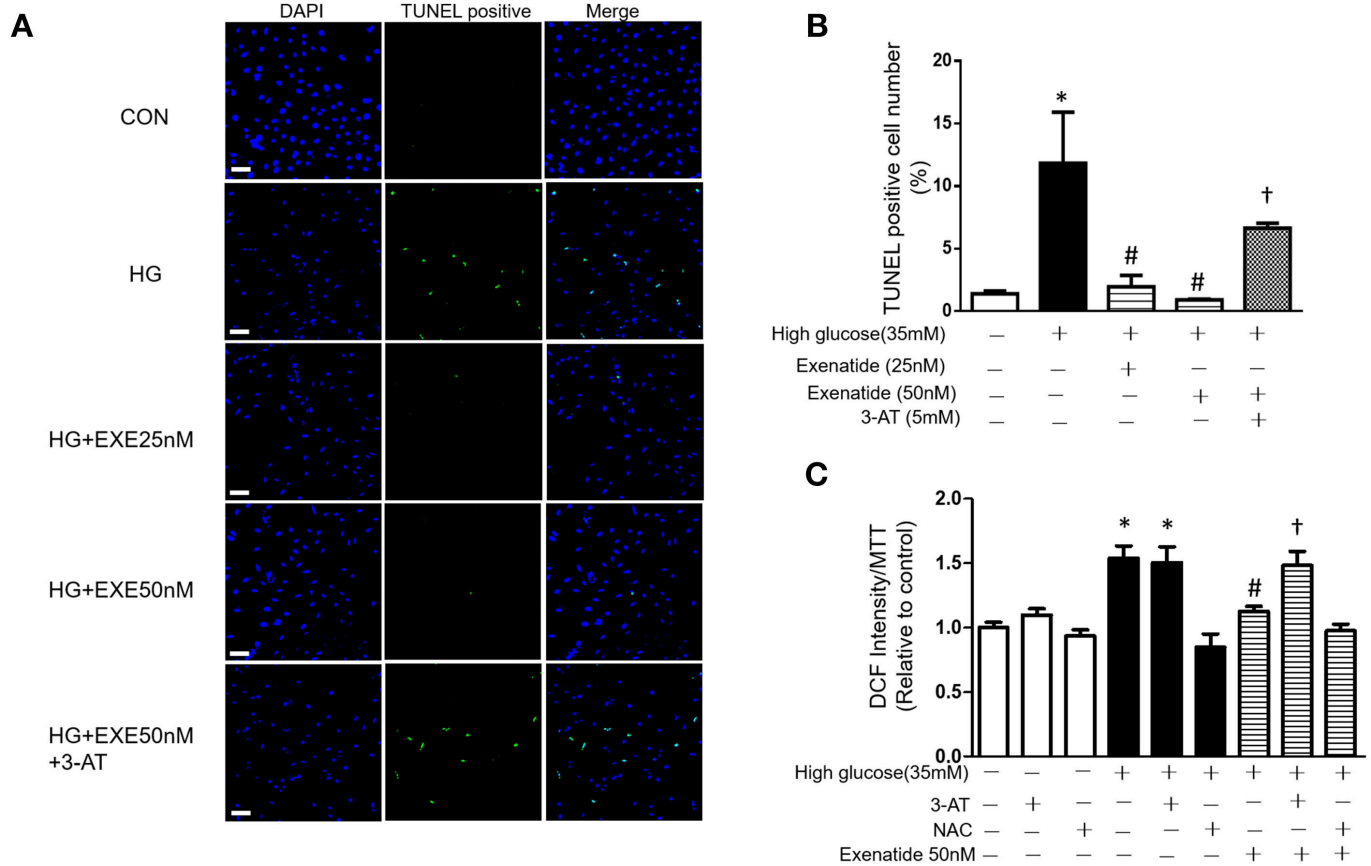
Exenatide treatment attenuates high glucose-induced apoptosis in H9c2 cells. H9c2 cells were incubated in the absence or presence of high glucose in combination with exenatide or the catalase inhibitor 3-AT or both. Cell apoptosis was measured by TUNEL assay as described in the Materials and Methods: green, TUNEL-positive myocyte nuclei; blue, DAPI-stained nuclei. Representative TUNEL staining is depicted, white scale bar, 50 μm **(A)**, quantitative analysis of apoptosis **(B)**. ROS production was measured by fluorescence intensity at 488/525 nm **(C)**. Data are shown as the mean ± S.E.M. The data represent at least three separate experiments, and each group has at least 3 samples. ^*^p < 0.05 vs. control group, ^#^*p* < 0.05 vs. high-glucose group, ^†^*p* < 0.05 vs. high-glucose plus exenatide group.

We next examined cell apoptosis in high glucose-treated cells. H9c2 cells were incubated in the absence or presence of 35 mM glucose in combination with exenatide or the catalase inhibitor 3-AT or both. H9c2 cells incubated with 35 mM glucose exhibited in a significant increase in cell apoptosis compared to the control cells (Figure 4A). Treatment of H9c2 cells with exenatide (25 or 50 nM) attenuated the observed apoptosis in cells induced by high glucose. Further, incubation of high glucose-treated cells with exenatide (50 nM) in the presence of 3-AT resulted in an elevation of cell apoptosis compared with exenatide treatment alone (Figures 4A,B). Thus, exenatide treatment attenuates high glucose-induced cell apoptosis in H9c2 cells.

**Figure 5:**
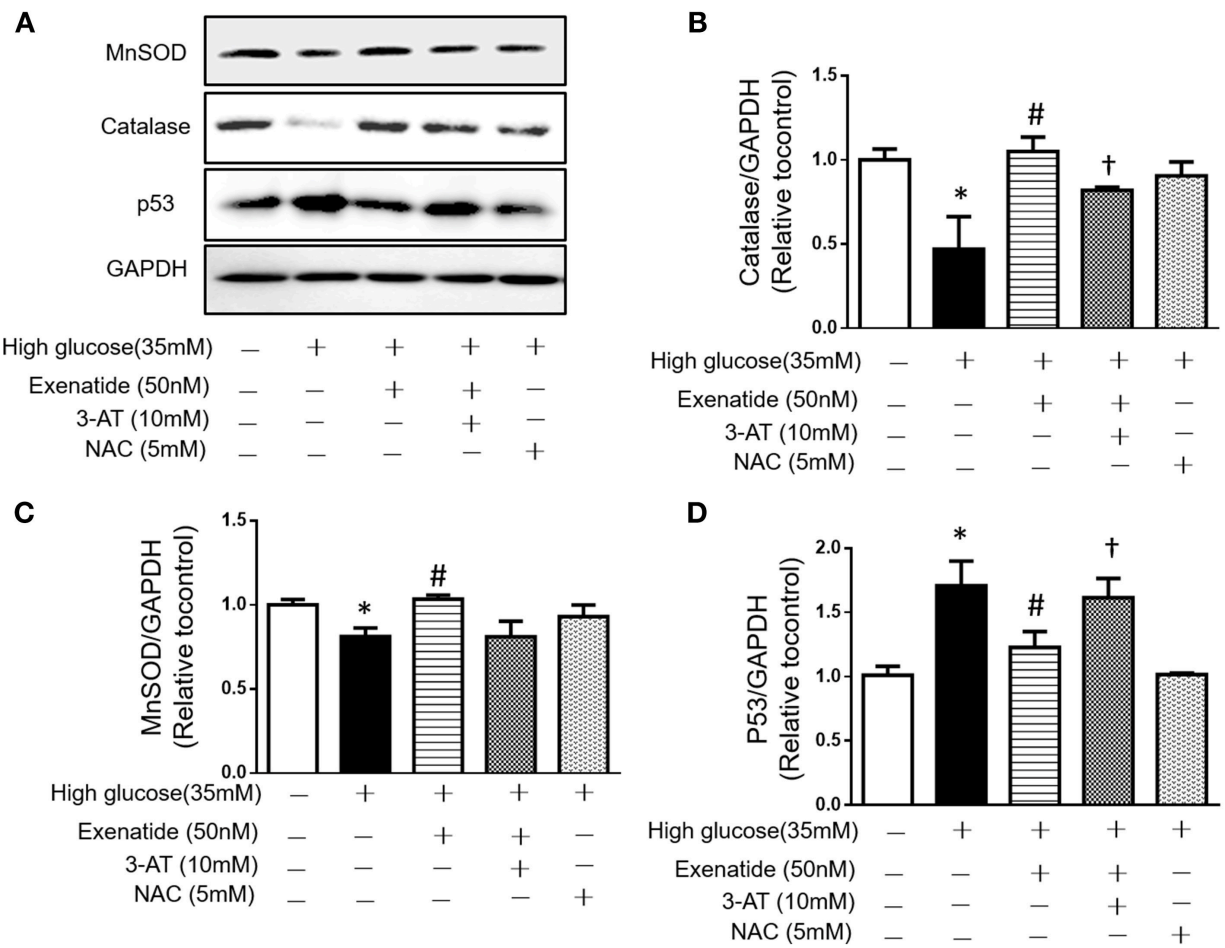
Exenatide prevents cell apoptosis by increasing catalase in H9c2 cells incubated with high glucose. H9c2 cells were incubated in the absence or presence of high glucose in combination with exenatide or the catalase inhibitor 3-AT or both, and the expression of catalase, MnSOD and p53 was determined as described in the Materials and Methods. Representative western blots are depicted **(A)**, catalase expression **(B)**, MnSOD expression **(C)**, and p53 expression **(D)**. Data are shown as the mean ± S.E.M. The data represent at least three separate experiments, and each group has at least 3 samples. ^*^p < 0.05 vs. control group, ^#^*p* < 0.05 vs. high-glucose group. ^†^*p* < 0.05 vs. high-glucose+Exenatide group.

Since high glucose-induced cell apoptosis results from an increase in ROS, we examined whether inhibiting antioxidant capacity could reverse this process. H9c2 cells were incubated in the absence or presence of 35 mM glucose in combination with exenatide or the catalase inhibitor 3-AT or both. As shown in Figure 4C, incubation of H9c2 cells with high glucose increased ROS production compared to the control treatment. Treatment of high glucose-incubated cells with exenatide decreased ROS compared to the control treatment (Figure 4C). Cells coincubated with high glucose and 3-AT displayed in a further increase in ROS levels than cells treated with high glucose alone. The presence of 3-AT attenuated the exenatide-induced reduction in ROS in high glucose-incubated cells (Figure 4C). The antioxidant protein expression levels were consistent with ROS production. Incubation of H9c2 cells with high glucose decreased MnSOD and catalase compared to the control condition (Figures 5A–C). The exenatide-induced elevation of catalase was attenuated by 3-AT. The reduction in p53 by exenatide treatment was also diminished by 3-AT (Figures 5A,B,D), indicating that exenatide exerts its antiapoptotic effects via elevating MnSOD and catalase levels, thus attenuating ROS production.

## Discussion

In the present study, we found that exenatide protected cardiac function in both type 1 and type 2 diabetic mice. In addition, exenatide simultaneously attenuated ROS production through increased antioxidant enzyme levels, which were observed both in mouse hearts and H9c2 cardiomyocytes. Moreover, inhibition of catalase by a catalase inhibitor (3-AT) diminished exenatide's protective effect on cell survival. These observations suggested that regulation of ROS production may be involved in the exenatide-mediated improvement of cardiac function in diabetic mouse hearts.

The T1DM and T2DM mouse models we used in the current study are two well-developed diabetic mouse models and have been used extensively in studies related to diabetes (23–25). T1DM is characterized by impaired beta cells and decreased insulin secretion. STZ was used as a method of β-cell ablation. This model exhibits extremely high glucose levels due to insufficient insulin secretion by islets (23, 26, 27). High-fat diet-induced type 2 diabetes is characterized by obesity and insulin resistance. The high-fat diets used in this study contain a high content of saturated fatty acids (45% kcal of fat, mainly containing lard and soy oil). This kind of model is extensively used in studies of obesity, hepatic steatosis, type 2 diabetes and type 2 diabetes-related complications (28–30). This model exhibits normal or mildly elevated FBG levels with significant insulin resistance.

The doses of exenatide we chose in this study are based on previous studies. In humans, clinical administration of exenatide (10 μg) twice daily or exenatide (2 mg) once weekly as an add-on therapy to type 2 diabetic patients was associated with greater improvements in several cardiovascular risk markers (7, 31–33). In animal studies, the doses of exenatide are different according to the aim of the therapeutic target. Exenatide (500 μg/kg) administered daily for 9 months promoted beneficial effects on short- and long-term memory performance in presenilin-1 knock-in (PS1-KI) mice, a preclinical model of amyloid-independent neuronal dysfunction, and exenatide (500 μg/kg) was administered daily for 2 months to delay age-dependent cognitive decline in normal adult mice (34, 35). A molecular mechanism study of intestinal growth used exenatide (42 μg/kg, twice daily, equal to 10 nmol/kg) as a GLP-1R agonist (36). In a T2DM mouse model induced by a high-fat diet, 20 μg/kg/d exenatide (equal to 4.8 nmol/kg/d) or 100 μg/kg/d exenatide (equal to 24 nmol/kg/d) was used to investigate its effects on the mechanisms of vascular aging in atherosclerosis or hepatic steatosis (37–40). Further, studies using exenatide (24 nmol/kg/d) to reverse the cardiac and cognitive dysfunction observed in high-fat diet-induced T2DM mice (41, 42). As our study used a similar model, we chose 24 nmol/kg/d for the dose of exenatide as a therapeutic agent for both T1DM and T2DM mice.

Exenatide was reported to protect cardiac function by anti-inflammation or autophagy upregulation (43). Additionally, exenatide attenuates cardiac hypertrophy and prevents cardiac apoptosis via AMPK activation (44, 45). Therefore, exenatide's cardioprotective effects may be related to multiple signaling pathways. Oxidative stress and the accelerated ROS production induced by high glucose are known to play key roles in the progression of diabetic cardiovascular disease and cardiomyocyte apoptosis. High plasma glucose-induced oxidative stress, typified by ROS production, promotes increased damage to DNA, RNA, proteins, mitochondria and cell membranes, triggering a series of maladaptive stimuli that result in alterations in signal transduction and gene expression that can lead to cell death (46, 47). The key initial step in ROS formation is the conversion of molecular oxygen (O_2_) to superoxide (O${}_{2^{-}}$). Superoxide dismutases (MnSOD and ZnSOD) are key enzymes that neutralize O${}_{2^{-}}$ into less reactive hydrogen peroxide (H_2_O_2_), which is then reduced to H_2_O by catalase or glutathione peroxidase. In this case, the intracellular antioxidative ability to increase ROS levels is largely determined by the expression of the enzymes SOD, catalase, and glutathione peroxidase (GSH-Px). It has been reported that exenatide treatment inhibits ROS production and attenuates fibrotic islet destruction in apoptotic pancreatic beta cells induced by fatty acids (48). In our study, we further found that in diabetic mouse heart and high glucose-stimulated cardiac cells, exenatide attenuated ROS production by increasing catalase and MnSOD expression, suggesting that the induction of antioxidant enzymes may underlie exenatide's protective effects.

P53 is closely related to cardiac cell apoptosis. Activation of p53 reduces the expression of genes opposing cell death, such as Bcl-2, and upregulates genes promoting apoptosis, such as Bax, therefore promoting cell apoptosis (49). In diabetic mice, suppressing p53 and the Bax/Bcl-2 ratio prevented progenitor cell apoptosis (50). P53 is also known to be induced by ROS (51). Antioxidant enzymes also affect p53 expression, and specific kidney overexpression of catalase reduces p53 levels and prevents apoptosis in mouse renal proximal tubules (52). In our present study, we found increased expression of p53 in diabetic mouse hearts, and exenatide treatment reduced p53 levels and increased catalase and MnSOD levels, suggesting that the antiapoptotic effects of exenatide is mediated, at least in part, by a reduction in ROS.

In summary, our current findings demonstrate that exenatide treatment improves cardiac dysfunction in diabetic mice and attenuates apoptosis in high glucose-induced H9c2 cardiomyoblasts. The mechanisms underlying these beneficial effects are a reduction in ROS production via increased activation of MnSOD and catalase and reduced P53 activation. We hypothesize that exenatide may be a candidate drug for the treatment of diabetic-related cardiovascular disease.

## Ethics Statement

This study was carried out in accordance with the recommendations of the tips in the Guide for the Care and Use of Laboratory Animals of the Institutional Animal Care and Use Committee, China. The protocol was approved by the Ethics Committee for the Use of Experimental Animals of Qingdao University.

## Author Contributions

JW and XZ designed the study. WD, WC, and JW drafted and wrote the manuscript. WD, WC, DX, XG, YL, and DD performed the experiments and statistical analyses. All authors have read and approved the final version of the manuscript.

### Conflict of Interest Statement

The authors declare that the research was conducted in the absence of any commercial or financial relationships that could be construed as a potential conflict of interest.

## Abbreviations

ROS: reactive oxidative species
GLP-1: glucagon-like peptide-1
IPGTT: intraperitoneal glucose tolerance test
MnSOD: manganese-dependent superoxide dismutase
MAPK: mitogen-activated protein kinases
P53: tumor protein P53.
